# The dead seed coat functions as a long-term storage for active hydrolytic enzymes

**DOI:** 10.1371/journal.pone.0181102

**Published:** 2017-07-11

**Authors:** Buzi Raviv, Lusine Aghajanyan, Gila Granot, Vardit Makover, Omer Frenkel, Yitzchak Gutterman, Gideon Grafi

**Affiliations:** 1 French Associates Institute of Agriculture and Biotechnology of Drylands, The Institutes for Desert Research, Ben-Gurion University of the Negev, Midreshet Ben-Gurion, Israel; 2 The Zuckerberg Institute for Water Research, The Institutes for Desert Research, Ben-Gurion University of the Negev, Midreshet Ben-Gurion, Israel; 3 Department of Plant Pathology and Weed Research, ARO, The Volcani Center, Bet Dagan, Israel; University of Manitoba, CANADA

## Abstract

Seed development culminates in programmed cell death (PCD) and hardening of organs enclosing the embryo (e.g., pericarp, seed coat) providing essentially a physical shield for protection during storage in the soil. We examined the proposal that dead organs enclosing embryos are unique entities that store and release upon hydration active proteins that might increase seed persistence in soil, germination and seedling establishment. Proteome analyses of dead seed coats of Brassicaceae species revealed hundreds of proteins being stored in the seed coat and released upon hydration, many are stress-associated proteins such as nucleases, proteases and chitinases. Functional analysis revealed that dead seed coats function as long-term storage for multiple active hydrolytic enzymes (e.g., nucleases) that can persist in active forms for decades. Substances released from the dead seed coat of the annual desert plant *Anastatica hierochuntica* displayed strong antimicrobial activity. Our data highlighted a previously unrecognized feature of dead organs enclosing embryos (e.g., seed coat) functioning not only as a physical shield for embryo protection but also as a long-term storage for active proteins and other substances that are released upon hydration to the “seedsphere” and could contribute to seed persistence in the soil, germination and seedling establishment.

## Introduction

The seed coat is a major defense against harmful environmental conditions protecting the embryo from mechanical stress as well as from microorganism invasion and from temperature and humidity fluctuations during storage. The seed coat is of maternal origin and is derived from the integuments (inner and outer) surrounding the ovule. However, it is not clear whether the dead seed coat was evolved to provide just a passive, physical shield for embryo protection or may represent also an active entity that stores and releases upon hydration substances (proteins, metabolites) that aid in seed persistence and longevity, germination and seedling establishment.

During *Arabidopsis* seed development five cell layers can be distinguished in the seed coat. The outer two cell layers having noticeable vacuoles derived from the outer integument, while three cell layers are derived from the inner integument [1]; the middle cell layer of the inner integument only surrounds part of the embryo sac [2]. The innermost cell layer of the inner integument, also known as endothelium, becomes vacuolated soon after fertilization and accumulates pigments mostly pro-anthocyanidins (PAs) [3]. Mucilage starts to accumulate during the torpedo stage in the outer cell layer of the outer integument. In *A*. *thaliana*, castor bean and tomato seeds the endosperm is undergoing programmed cell death (PCD) commonly prior to occurrence of PCD in the integuments [4–6]. In Brassicaceae, the fate of the endosperm in mature seeds is variable. For example, mature seeds of *Raphanus* and *Brassica* are without endosperm, mature seeds of *A*. *thaliana* and *Lepidium* spp. retain a single cell/thin layer of live endosperm while Fabaceae species have cotylespermous seeds whereby the endosperm is short lived and not exist at maturation and cotyledons assume the function of storage tissue [reviewed in 7]. At maturity, all cell layers of the seed coat are dead; most cell layers are crushed together except for the epidermis, that is, the outer cell layer of the outer integument [8,9]. In *Arabidopsis*, as well as in other Brassicaceae species, the outer cell layer of the seed coat functions as a special type of secretory cells that synthesize large amounts of pectinaceous mucilage during seed development and maturation [10,11]. This mucilage is rapidly swelled during hydration generating a gelatinous capsule around the seed that functions in seed dispersal and adhesion to soil as well as providing a water reservoir for increasing success of germination, particularly under water scarcity [12–14]. In addition, seed coat mucilage may be important for maintaining the DNA repair mechanism within the embryo and thus assists in seed viability and consequently in maintaining a functional seed bank in hostile environments [15,16]. There are reported data implicating mucilage as a barrier regulating diffusion of water and oxygen to the inner tissues to prevent germination under inappropriate conditions [14]. Notably, *Arabidopsis* mucilage mutants, including *ttg1*, *gl2*, *atsbt1*.*7* and *dcr-1* germinated normally under non-stressed conditions but displayed reduced germination and seedling establishment in the presence of polyethylene glycol [17–19].

Perhaps, seed germination represents the most vulnerable stage of plant development as seeds are germinated into a potentially hostile, stressful environment. Furthermore, during storage in the soil, seeds are often subjected to vulnerable biotic and abiotic conditions (microbe attack, humidity and temperature fluctuations), which could affect longevity and persistence of seeds. Yet, seeds of many plant species persist in the soil and maintain viability for many years [20]. The mechanisms underlying seed persistence and viability in the soil have been addressed mostly with respect to chemical defense (secondary metabolites). For example, examination of seeds of over 80 plant species from the British flora and of some agricultural important weeds revealed that seeds contain at least traces of hydroxyphenols and many of them released hydrogen cyanide upon hydration, which might provide a defense layer against microbes and seed herbivores [21]. Seeds of various plant species such as *Raphanus sativus*, *Vigna unguiculata* (cowpea), *Phytolacca americana and Mirabilis jalapa* were found to contain and secrete various proteins including β-1,3-glucanases and small, anti-fungal proteins (AFPs) with strong activity against fungal pathogens and Gram-positive bacteria [22–24]. Yet, it is not clear if the origin of these substances is maternal (e.g., seed coat) or zygotic (i.e., embryo). Notably, pigments in the maternally-derived seed coat that resulted from production of phenolic compounds (e.g., tannins) are often associated with antioxidant content and defense activity against pathogens [25–27]. It should be mentioned that proteome analysis of live seed coats was performed for developing seeds of soybean (*Glycine max* L.) revealing multiple proteins including an abundant protein of about 32 kDa class I chitinase [28] that plays a role in plant defense against pathogens [29–30].

Recently, proteome analysis of the dead floral bracts of the dispersal unit of wild emmer wheat (i.e., glumes) revealed that many proteins are stored and released upon hydration including S1-type nucleases, peptidases, antifungal hydrolases such as chitinases and β-1,3-glucanase as well as reactive oxygen species (ROS)-detoxifying enzymes such as superoxide dismutase and ascorbate peroxidase [31] (Raviv et al., 2017). Yet, no data exist on the role of dead seed coats in storing of proteins, their composition and their endurance in active forms. We hypothesized that similarly to dead glumes, the maternally-derived seed coats store proteins and other substances (e.g., antimicrobial substances, nutrients) that are released to the seed’s immediate surroundings (‘seedsphere’) upon hydration and assist in seed persistence and longevity, germination and seedling establishment. We report here that seed coats of *Sinapis alba* and *Anastatica hierochuntica* store and release upon hydration hundreds of proteins including proteins involved in stress response, many function as hydrolases such as nucleases, proteases and chitinases. In gel assays demonstrated that hydrolases can persist for decades in active forms within the dead seed coats. Thus, our data highlighted the potential function of the dead seed coat in storing and releasing upon hydration multiple substances that might explain, at least partly, how seeds persist and retain viability in the soil for many years.

## Materials and methods

### Plant materials, secretion of substances and mucilage staining

Seeds of various wild crucifers and leguminous species were collected in the field in Israel or obtained from the Israel Plant Gene Bank (S1 Table). No specific permissions were required for all locations from which seeds were collected. These locations include Road sides in central and northern Negev or the campus area at Midreshet Ben Gurion. Seeds of *Arabidopsis thaliana* (Col and Ler) wild type and mucilage mutant plants *gl2* and *mum4* (kindly provided by T. Western and S. Harpaz-Saad) were collected from plants grown in growth room at 22°C±2°C under long day photoperiod.

Analysis of substances released from seeds of various plant species was standardized either based on seed weight or on seed surface area. Briefly, 10 mg of seeds were incubated in 100 μl of phosphate-buffered saline (PBS) at 4°C for 8–12 h after which the aqueous phase was collected by centrifugation (4°C, 11,000 rpm, 5 min) and the supernatant was used immediately or stored at -20°C until used. Because, the seeds examined in this study have various volume, shape and weight, in some experiments, standardization was based on surface area of seeds equivalent to 10 mg of *Arabidopsis* seed surface area. Seeds examined in this study were either ellipsoid in shape (e.g., *Arabidopsis thaliana*) or round (e.g., *Sinapis alba*), and therefore we calculated the surface area of ellipsoidal seeds by the equation: SA = 4π/(a^p^b^p^+a^p^c^p^+b^p^c^p^)/3]^1/p^ where SA is the surface area, a, b and c are the semi-principal axes of the ellipsoidal seed, π = 3.14 and p = 1.6, while the surface area of round seeds by the equation SA = 4πr^2^, where SA is the surface area, π = 3.14 and r, is the radius of the seed. All calculations were performed using on line calculators (https://planetcalc.com/149/). Accordingly, the surface area of 10 mg *Arabidopsis* seeds is calculated by the surface area of one seed (~0.4 mm^2^) multiply by the number of seeds in 10 mg (~400 seeds). Thus, the number of seeds attaining the same surface area of other Brassicaceae species was calculated by dividing the surface area of 10 mg *Arabidopsis* seed (~160 mm^2^) by the surface area of one seed of a given Brassicaceae species. Finally, in experiments where comparison were performed between seeds, embryos and seed coats we use a given number of seeds from which embryos and seed coats were dissected.

Mucilage was stained with Ruthenium red essentially as described [32]. Seeds were hydrated with either distilled water or 50 mM EDTA for 90 min, washed with distilled water and incubated with 0.01% Ruthenium Red (Sigma-Aldrich) 90 min. The seeds were washed with distilled water to remove access dye and observed under a binocular microscope (Zeiss).

### Proteome analysis

Proteome analysis of seeds and seed coats were performed by the proteomic services of The Smoler Protein Research Center at the Technion, Israel. Proteins released from 10 mg *Arabidopsis* seeds or from 2 mg seed coats of *Sinapis alba* and *Anastatica hierochuntica* following hydration (4°C, 12 h in PBS) were digested with trypsin followed by separation and mass measurement on LC-MS/MS on LTQ-Orbitrap and identification by Discoverer software software against the uniprot database, which contains plants and fungi proteins and against a decoy database in order to determine the false discovery rate. All the identified peptides were filtered with high confidence, top rank, mass accuracy, and a minimum of 2 peptides. High confidence peptides were passed the 1% FDR threshold (FDR = false discovery rate, is the estimated fraction of false positives in a list of peptides). At least 2 replicates were performed for each examined organs. Semi-quantitation was done by calculating the peak area of each peptide. The area of the protein was calculated from the average of the two to three most intense peptides from each protein. All proteome parameter definition are given in S2 Table.

We applied a more stringent filtering to the proteins, which are considered present in each plant. Considering that the average amino acid length of the proteins in the dataset was nearly 400 and assuming that 2 peptides are the minimum requirement for an average protein, we extrapolated this cutoff to consider protein length as the following: The minimum number of peptides per protein is the protein length divided by 200, but not less than 2 peptides. Additionally, the protein coverage by peptides from all samples must be higher than 10% for the Arabidopsis seed secretion proteome data (S1 data) and 20% for the analysis of seed coats of *Sinapis alba* and *Anastatica hierochuntica* (S2 data). Therefore, a protein is regarded as “present” in a sample if the number of peptides is above the threshold for that protein length, the signal is >0, and the total peptide coverage is either >10% or >20%. A protein is regarded as “present” in a plant if it is “present” in at least two replicates of that plant.

GO categorization analysis was carried out using the BiNGO (v3.0.3) plugin of Cytoscape (v3.4.0). GO ontology file (go.obo) was downloaded from geneontology.org (http://purl.obolibrary.org/obo/go.obo). Annotation of UniProt accessions with GO terms was according to UniProt. For the reference set we used the 246 proteins, which were present in at least one of the plants. No statistical test for GO enrichment was carried out, and only the number of proteins in each GO term was counted. The following GO trees were examined, Biological Process (BP), Molecular Function (MF) and Cellular Component (CC). GO Slim for plants, which included broad categories of BP, MF and CC was also performed and the results were sorted by descending number of proteins per GO term.

### In-gel nuclease assay

Nuclease assay was performed essentially as described [33] in polyacrylamide gel containing 300 μg/ml denatured salmon sperm DNA or ribonucleic acid from Torula yeast (Sigma) for RNases activity. Briefy, proteins released from seeds, embryos or seed coats were incubated with sample buffer containing 2% SDS, 62.5mM Tris pH 6.8 and 10% glycerol and bromophenol blue for 1h at 37°C followed by separation on SDS/PAGE (samples were not boiled). The gel was washed twice, each time for 30 min, at room temp in buffer containing 10 mM Tris-HCl pH 7.5 and 25% isopropanol, followed by washing twice 15 min each with 10 mM Tris-HCl pH 7.5. Nuclease activity was performed by incubating the gel with 10 mM Tris-HCl pH 7.5 with or without divalent cations. We used either combination of 10 mM MgSO_4_ and 10 mM CaCl_2_ or only 10 mM ZnCl_2_ as cofactors for 75 min at 37°C. The gel was stained for 60–80 minutes with 10 mM Tris HCl pH 7.5 containing 2 μg/ml ethidium bromide and inspected under UV light.

### Endonuclease conversion assay

S1-type endonuclease activity was assessed by the conversion assay, that is the capability to convert supercoiled plasmid DNA into relaxed and linear forms essentially as described [34]. Briefly, 1 μg of supercoiled plasmid DNA was incubated with substances released from seeds in buffer containing 10 mM Tris-HCl pH 7.5, 10 mM MgSO_4_ and 10 mM CaCl_2_. Samples were incubated at room temperature for various time periods and reactions were stopped by adding EDTA to a concentration of 50 mM. The conversion of supercoiled plasmid DNA into relaxed and linear forms was monitored after separation on 1% agarose gel containing ethidium bromide.

### In-gel chitinase assay

In gel chitinase assay was performed essentially as described [35]. Briefly, Intact seeds of *Raphanus sativus*, seed coats and isolated embryos were incubated in 0.1M NaHPO4 (pH 6) at 4°C for 16 h, after which the sup was collected and secretion from 2 seeds equivalent were taken for separation on SDS/PAGE. Samples were first incubated in chitin sample buffer (15% sucrose, 2.5% SDS, 12.5 mM Tris-HCl pH 6.7, 0.01% Bromophenol Blue) for 1 h at 37°C and samples were run on 12% SDS/PAGE containing 0.01% glycol chitin. The gel was incubated in buffer containing 100 mM sodium acetate (pH = 5.2) and 1% triton x-100 for 2 h at 37°C followed by staining for 5 min with 0.01% calcofluor white in 500 mM Tris-HCl (pH = 8.9). The gel was washed with distilled water for 1 h and visualized by UV transillumination.

### Antimicrobial assays

The spectrophotometric bioassay for microbial sensitivity was performed essentially as described [36]. We used *Staphylococcus aureus* as a representative model for Gram-positive bacteria. One colony of each strain was suspended in 10 ml of LB Broth (Difco, MD, USA) and grown overnight at 37°C. The cultures were diluted, transferred to 25% LB Broth and grew at 37°C to 0.03–0.05 optical density (OD_595_; Epoch, Biotek, USA). A 150 μl aliquot of the culture was incubated with 50 μl of PBS, ampicillin (final concentration 100 μg/ml) or with 50 μl of substances released from whole seed, embryo, or the seed coat (3–9 replicates per treatment) in a flat-bottom 96-well microtiter plate. Plates were incubated in the dark using a spectrophotometer (Synergy 4, Biotek, USA) and reads (OD_595_) were taken at intervals of 30 minutes in a course of 24 hours. The average OD for each blank replicate at a given time point was subtracted from the OD of each replicate treatment at the corresponding time point and standard errors were calculated for each treatment at every time point.

### Fungi spore germination assay

Microconidia of the pathogenic fungi *Fusarium oxysporum* f.sp. *melonis* as a model pathogen were obtained from two-week-old mycelium cultivated on potato dextrose agar plates and diluted in double distilled water to a level of 10^4^ microconidia per ml. Substances released from seeds (in PBS) were added to potato dextrose broth in a 1:1 ratio, and 63 μl of the mixture was vortexed with 7 μl of spore suspension and co-cultivated on a depression slide in a dark, moist chamber at 25°C. Conidial germination was monitored after 24 h under light microscope (Zeiss, Germany).

## Results

### Seeds release multiple proteins upon hydration—Proteome analysis

To gain insight into proteins released from seeds upon hydration and might contribute to seed germination and seedling establishment, we initially incubated seeds of *Arabidopsis thaliana* (Col) in PBS buffer at 4°C for 12 h and the supernatant was collected and subjected to proteome analysis using LC-MS/MS followed by identification by Discoverer software against the Uniprot database, which contains plants and fungi proteins; notably, only plant proteins were identified (S1 data). Implementing the cutoffs (at least 2 peptides and 10% coverage) we identified 238 proteins that were released from Arabidopsis seeds following hydration (S1 data). Functional classification using PANTHER classification system (Mi *et al*., 2005) revealed a high proportion of proteins with catalytic activity (55.2%), while among protein classes, hydrolases (18.5%), oxireductases (17.3%) and nucleic acid binding proteins (17.9%) were prominent. Classification for biological processes showed that 41.2% of the released proteins are involved in metabolic processes and 5.7% of the proteins are related to plant response to stimulus (Figure A in S1 File). Proteins responsive to stimulus include several plant defensin-like (DEFL) molecules also known as low molecular weight cysteine-rich (LCR) proteins encoded by At1g75830/AtPDF1.1, At2g12475/DEFL112 and At1g13607/DEFL286, as well as LCR17 (At4g11760) and LCR25 (At4g29305), which are implicated in defense response to fungus [37–39]. Other stress-related proteins include the pathogenesis related protein 5 (PR5, AT1g75040) and a thaumatin-like protein involved in response to pathogens [40]. Among hydrolases we identified multiple proteases and nucleases including several endopeptidases such as aspartyl proteases (At1g03220 and At3g54400), cysteine proteinase (At1g06260) and subtilisin-like serine endopeptidase (At5g03620), endonuclease BFN1/ENDO1 (At1g11190) whose expression was studied with respect to senescence and cell death [41] and THIOGLUCOSIDE GLUCOHYDROLASE 1 (AtTGG1, At5g26000), an enzyme that catalyzes the hydrolysis of glucosinolates into compounds that are toxic to various microbes and herbivores [42].

Notably, identification of proteins was performed by the Discoverer software against the Uniprot database, which contains plants and fungi proteins, confirming that proteins released from *Arabidopsis* seeds upon hydration (at 4°C) are of plant origin and not derived from microbes.

### Proteins released from seeds following hydration are enzymatically active

**Fig 1:**
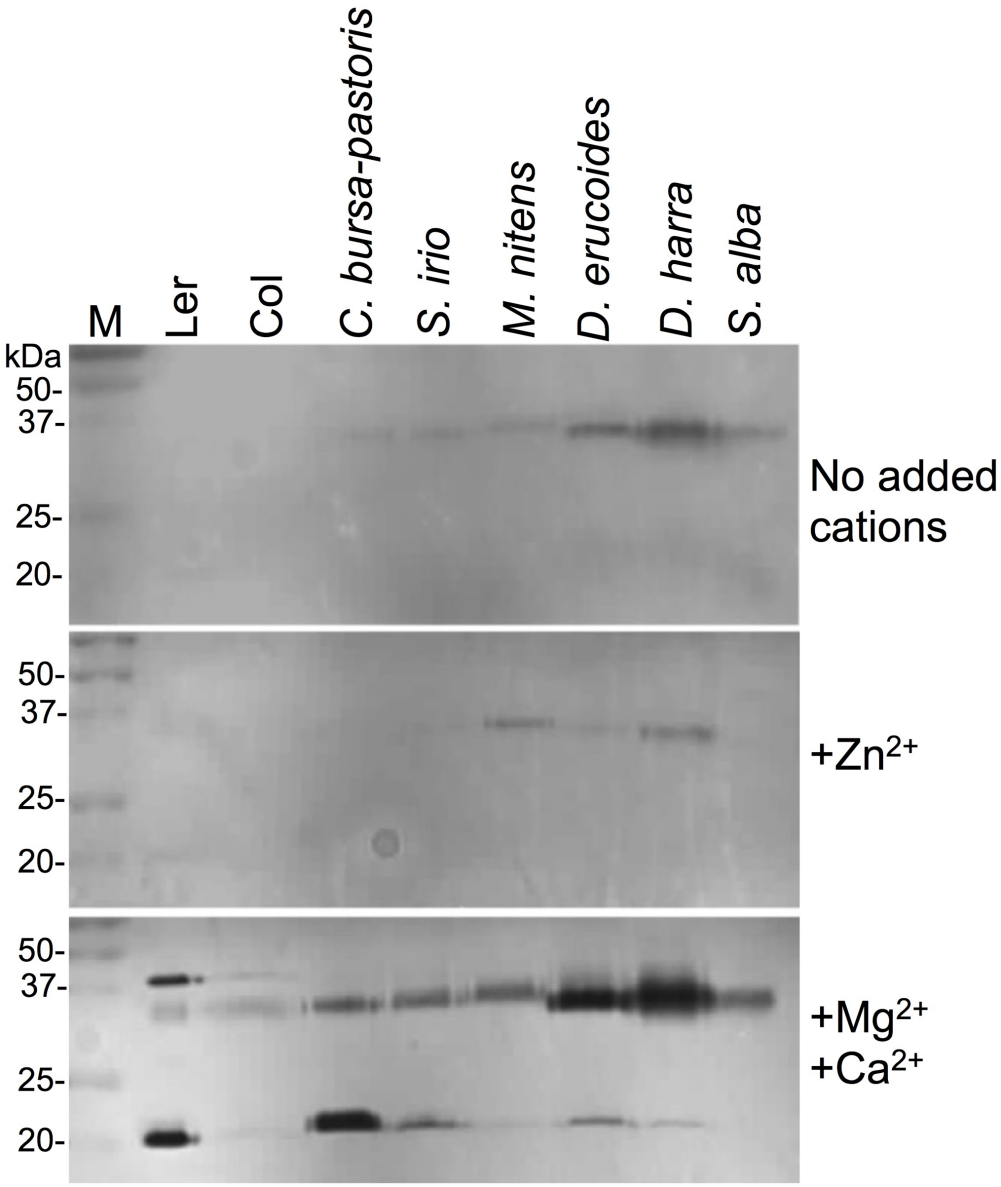
Nuclease activities in seed secretions of Brassicaceae species. In-gel nuclease assay demonstrating high nuclease activity in secretions from seeds of various crucifers. Seeds of *Arabidopsis thaliana* Ler and Col lines, *Capsella bursa-pastoris*, *Sysimbrium irio*, *Moricandia nitens*, *Diplotaxis erucoides*, *Diplotaxis harra* and *Sinapis alba* were incubated in PBS for 8 h at 4°C, the aqueous phase was collected and proteins were separated on 12% SDS/PAGE containing denatured salmon sperm DNA. Nuclease reaction was performed with or without the indicated divalent cations and activity was visualized by staining with ethidium bromide. M, protein size markers. Note that strong nuclease activity was recovered in the presence of Mg^2+/^Ca^2+^.

The possibility existed that some of the hydrolases recovered in the proteome analysis are partially degraded and are not enzymatically functional. One enzyme released upon hydration is BFN1/ENDO1 endonuclease implicated in senescence and PCD. We analyzed for endonuclease activity by in gel nuclease assays using denatured salmon sperm DNA as substrate and various cations as cofactors. Here we extended the analysis to measure nuclease activity in substances released from various mucilaginous seeds of Brassicaceae species. To this end, we collected in the field seeds from wild crucifers including *Capsela bursa-pastoris* (L.) Medik., *Sisymbrium irio* L., *Moricandia nitens* (Viv.) E. A. Durand & Barratte, *Diplotaxis erucoides* (L.) DC., *Diplotaxis harra* (Forssk.) Boiss. and *Sinapis alba* L. Results showed (Fig 1) that all examined seeds of wild species released nucleases upon hydration similarly to *Arabidopsis*. In all cases, no or very low activity was observed in the absence of cations or in the presence of Zn^2+^ but high nuclease activities at positions of about 22 and 35 kDa were recovered in the presence of Ca^2+^ and Mg^2+^.

### Most nucleases are released from the dead organs enclosing the embryo

**Fig 2:**
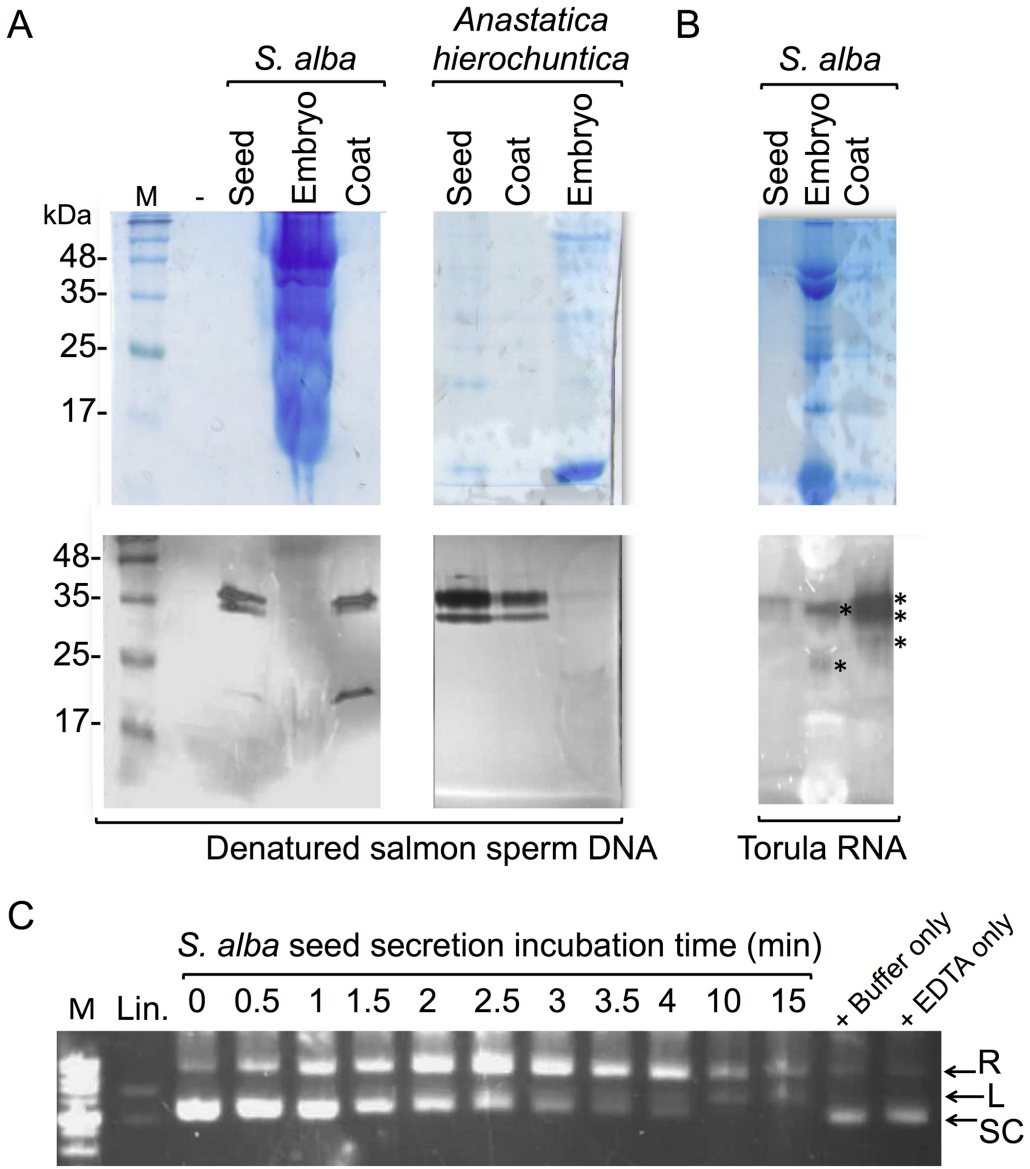
Nucleases are released from the seed coat. A, Proteins released from seeds, seed coats and embryos of the indicated Brassicaceae species were subjected to in gel nuclease assay using denatured salmon sperm DNA as substrate. Upper panels are the EZBlue staining and the lower panels are the nuclease assays. M, protein molecular weight markers. B, In gel RNase assay. Proteins released from seeds, seed coats and embryos of *S*. *alba* were subjected to in gel nuclease assay using Torula yeast RNA as substrate. Asterisks at the lower panel mark positions of active RNases. Upper panel is the EZBlue staining gel. C, In vitro endonuclease assay. Supercoiled plasmid DNA (1 μg) was incubated for the indicated time points at room temperature with *S*. *alba* seed secretion. Supercoiled plasmid DNA incubated with the buffer only or with 50 mM EDTA only, for 15 min, were included as controls. Lin is restriction enzyme linearized plasmid (marked by white asterisk). The positions of the different topological forms of plasmid DNA are indicated: R, relaxed form; L, linear; SC, supercoiled plasmid DNA. M, molecular size markers of 1 kb DNA ladder.

Nucleases released from the dry seed upon hydration could have been released from the embryo or from dead organs enclosing the embryo, namely, the seed coat. To test the origin from which nucleases are released, we separated seed coat from the embryo of three crucifer species having relatively large seeds, namely, *S*. *alba*, *D*. *harra* and *Anastatica hierochuntica* L. Separated seed coats and embryos were hydrated, the sup was collected and analyzed for nuclease activities using in gel nuclease assay. Results showed (Fig 2A) that nucleases were released from the seed coat of all examined species but not from the embryo itself. We also analyzed for the presence of ribonucleases in substances released from *S*. *alba* seed using in-gel assays with yeast Torula RNA as a substrate. Results showed (Fig 2B) the presence of several differentially migrating ribonucleases ranging from 27 to 37 kDa in substances released from both the embryos and seed coats; high RNase activities were recovered from the seed coat of *S*. *alba*.

The timing by which nucleases are released from seeds upon hydration was relatively very fast and occurs within 15 min following hydration; maximal nuclease activity was observed after 30 min of hydration (Figure B-A in S1 File). We also analyzed the dynamics of release of nucleases from seed coats of *S*. *alba* by repeated extraction with PBS. The Results showed that most nucleases were released in the first extraction round and then gradually decreased (Figure B-B in S1 File).

Although Brassicaceae seeds are exalbuminous, that is, the endosperm is consumed during embryo development and undergoes cell death, in certain species such as *A*. *thaliana* mature seeds may contain a single cell layer of the endosperm which is alive and often firmly associated with the dead seed coat [7]. To verify that proteins were released from the dead seed coat rather than from the endosperm, we also analyzed mature seeds of species that are deficient of endosperm such as Fabaceae species having cotylespermous seed storage tissue [7]. In most legumes, the endosperm is short-lived and it is absorbed during seed development and at maturity it is not present or is visible as a thin layer surrounding the cotyledons or the embryo [43,44]. We have selected seeds of three leguminous species, namely, *Lupinus pilosus* L., *Cicer arietinum* L. (cultivated chickpea) and *Colutea istria* Miller and the seed coats were separated from embryos and analyzed for nucleases released upon hydration. Results showed that all examined leguminous species store and release nucleases exclusively from the seed coat (Figure C in S1 File), further supporting the notion that the essentially dead seed coats enclosing the embryo in a variety of plant species store and release active nucleases upon hydration.

### S1-type endonucleases are released from the seed coat

The 35 kDa nuclease released upon hydration from seeds of *Arabidopsis* and other examined species is the predicted molecular mass of BFN1/ENDO1 (identified in the proteome data), a nuclease related to S1-type endonucleases. S1-type endonucleases, such as *Aspegillus* S1 nuclease and mung bean nuclease are widely used in molecular biology applications and are capable of introducing nicks and double strand DNA breaks (DSBs) into supercoiled plasmid DNA converting it to relaxed and/or linear forms—a well-established method for monitoring single-strand DNA endonucleases [45]. The capacity of single-stranded DNA endonucleases to target and change the topology of double-stranded supercoiled plasmid DNA is a consequence of torsional strain generated in superhelical DNA, which promotes local denaturation and unpairing essentially at weakly hydrogen-bonded regions [46]. To monitor for specific S1-type endonuclease activity we incubated supercoiled plasmid DNA with proteins released from *S*. *alba* seeds for various time periods (0.5 to 15 min) followed by agarose gel electrophoresis. Results showed (Fig 2C) that substances released from *S*. *alba* upon hydration possess S1-type endonuclease activity that gradually converted supercoiled plasmid DNA into a linear form in two distinguishable steps. The first is seen by the gradual accumulation of the relaxed form concomitantly with gradual disappearance of the supercoiled plasmid DNA, which is followed by a second step of accumulation of the linear form. Thus, substances released from seeds contain S1 type endonucleases capable of introducing double strand DNA breaks into superhelical DNA.

### Long-term persistence of active nucleases in dead seed coats

**Fig 3:**
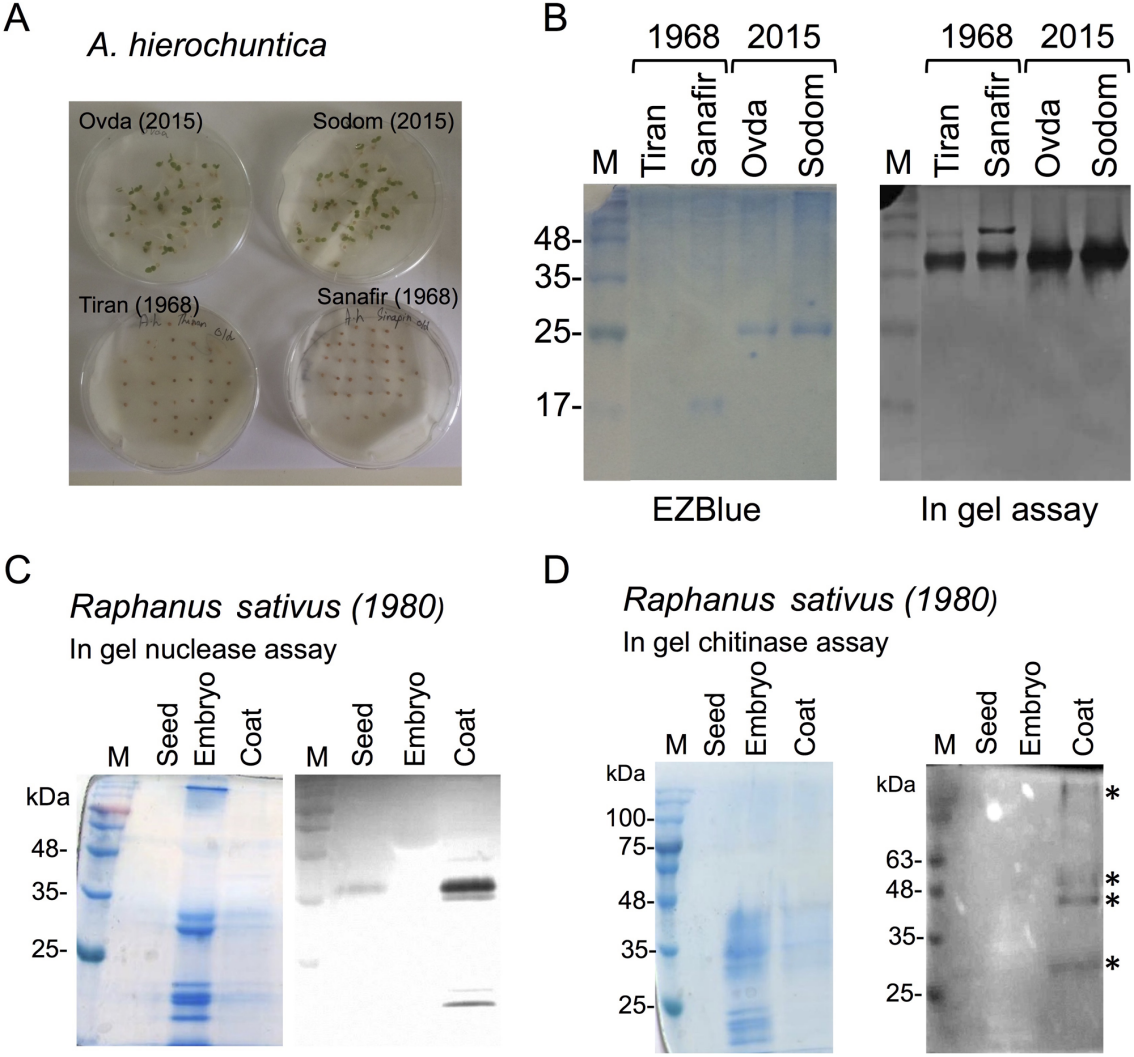
Long-term survival of nucleases within the dead seeds of *A*. *hierochuntica*. A, Germination test of new (2015) and old (1968) seeds of *A*. *hierochuntica* collected from various sites. Tiran and Sanafir ecotypes refer to islands located at the straits of Tiran that separate the Red Sea from the Golf of Aqaba/Eilat. Sodom ecotype was collected near Sodom mountain and the dead sea, while Ovda ecotype was collected near Eilat. B, Dead, non viable seeds of Tiran and Sanafir store and release upon hydration active nucleases. Seeds of the indicated ecotypes were incubated in PBS for 8 h, the aqueous phase was collected and analyzed by in-gel nuclease assay using denatured salmon sperm DNA as substrate. M is the protein molecular weight markers. Long-term survival of nucleases (C) and chitinases (D) within the seed coat of *Raphanus sativus* collected at 1980. 10 mg of intact seeds, seed coats and embryonic tissues were extracted with 100 μl PBS and one third of the extract was subjected to in gel assays. The gels in (C) and (D) were stained with EZblue following the nuclease and chitinase reactions. Asterisks in (D) indicate chitinases released from the seed coat. M, Protein molecular weight markers.

To test the endurance of nucleases within dead seed coats, we examined seeds of *A*. *hierochuntica* obtained from dried skeletons collected from the islands of Tiran and Sanafir at 1968 and stored at room temperature. Notably, when fruits matured, the *Anastatica* desiccated skeleton is rolled inward forming a ball shape structure that protect fruits and seeds from desiccation and predation, generating an aerial seed bank that remain viable for many years [47,48]. We first compared the germination capacity of 1968 seeds of Tiran and Sanafir ecotypes with those of 2015 seeds of Ovda and Sodom ecotypes. The results showed (Fig 3) no germination of the 1968 Tiran and Sanafir seeds compared to almost full germination of *A*. *hierochuntica* seeds collected during the year of 2015 (Fig 3A). Yet, both the 2015 and the 1968 seeds released upon hydration active nucleases (Fig 3B) suggesting that nucleases can be well preserved for decades in dead organs enclosing the embryos.

Further analysis of seeds of *Raphanus sativus* collected at 1980 (provided by Israel Plant Gene Bank) showed that all hydrolase activities, namely nucleases (Fig 3C) and chitinases (Fig 3D) are released from the dead seed coat providing further support that nuclease as well as chitinase activities persist within the dead organs for decades.

### Mucilage is not required for storage and release of hydrolases

**Fig 4:**
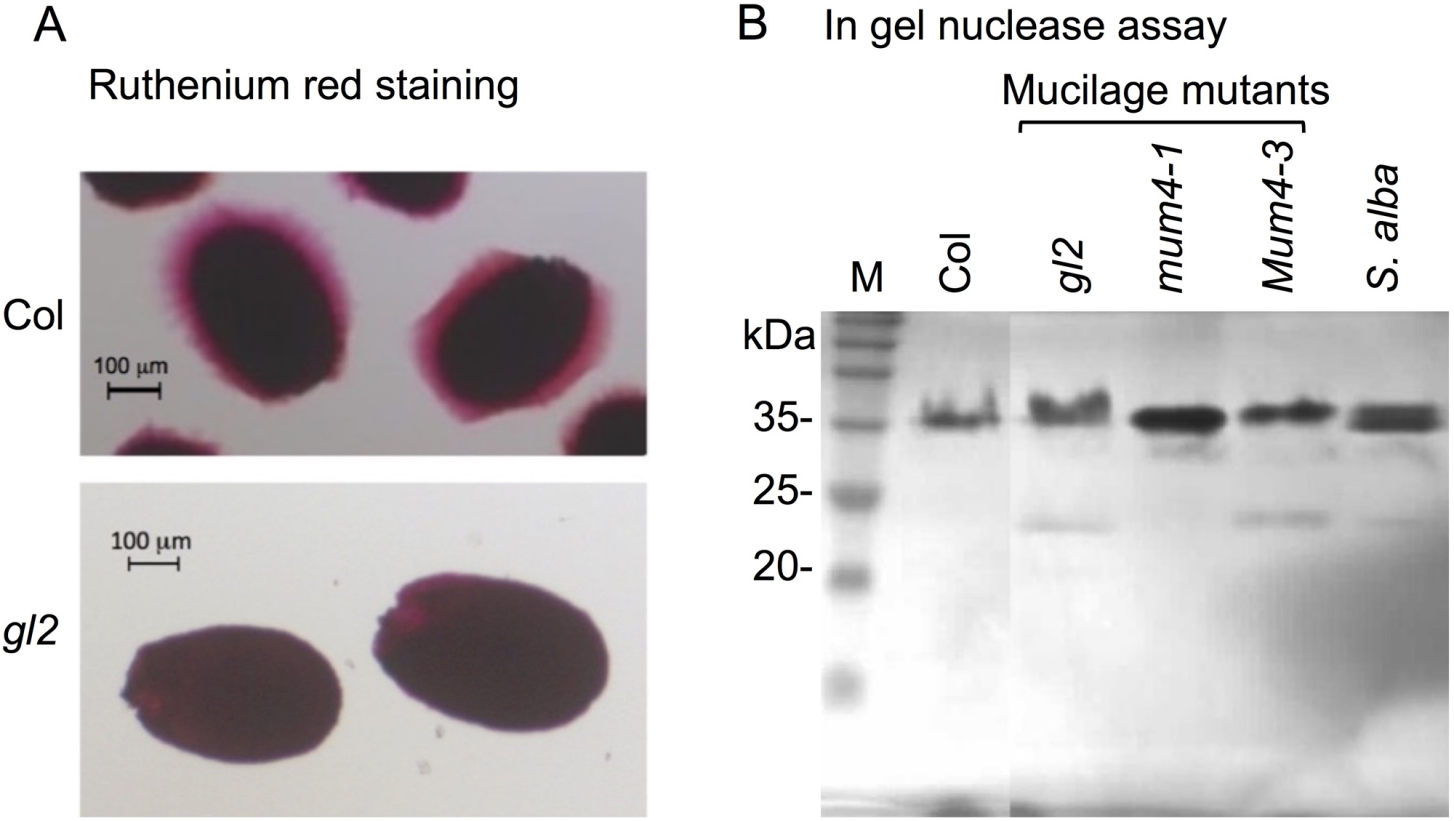
The seed coat mucilage is not required for storage and secretion of nucleases. A, Ruthenium red staining of *Arabidopsis* Col seeds and *gl2* mucilage mutant. B, In gel nuclease assay for proteins released from seeds of the indicated wild type and mucilage mutant lines. M, protein molecular weight markers.

Although many of the Brassicaceae species contain mucilage, *Raphanus sativus* as well as Fabaceae species are not suggesting that the mucilage is not an obligate requirement for storage and release of proteins. We wanted to test the importance of mucilage, in species naturally containing mucilage such as *Arabidopsis thaliana*, for storage and release of nucleases from the seed coat. To this end, we used several *Arabidopsis* mucilage mutants carrying mutation in genes whose products involved in the regulation of mucilage production, including GL2 and MUM4 [49]. Staining of seeds of Col and of the mucilage mutant *gl2* with ruthenium red demonstrated (as expected) the lack of mucilage in *gl2* (Fig 4A). However, in gel nuclease assay demonstrated (Fig 4B) that all mutant seeds released nucleases similarly to Col seeds.

### The dead seed coats of *S*. *alba* and *A*. *hierochuntica* store and release multiple proteins involved in stress response: Proteome analyses

**Fig 5:**
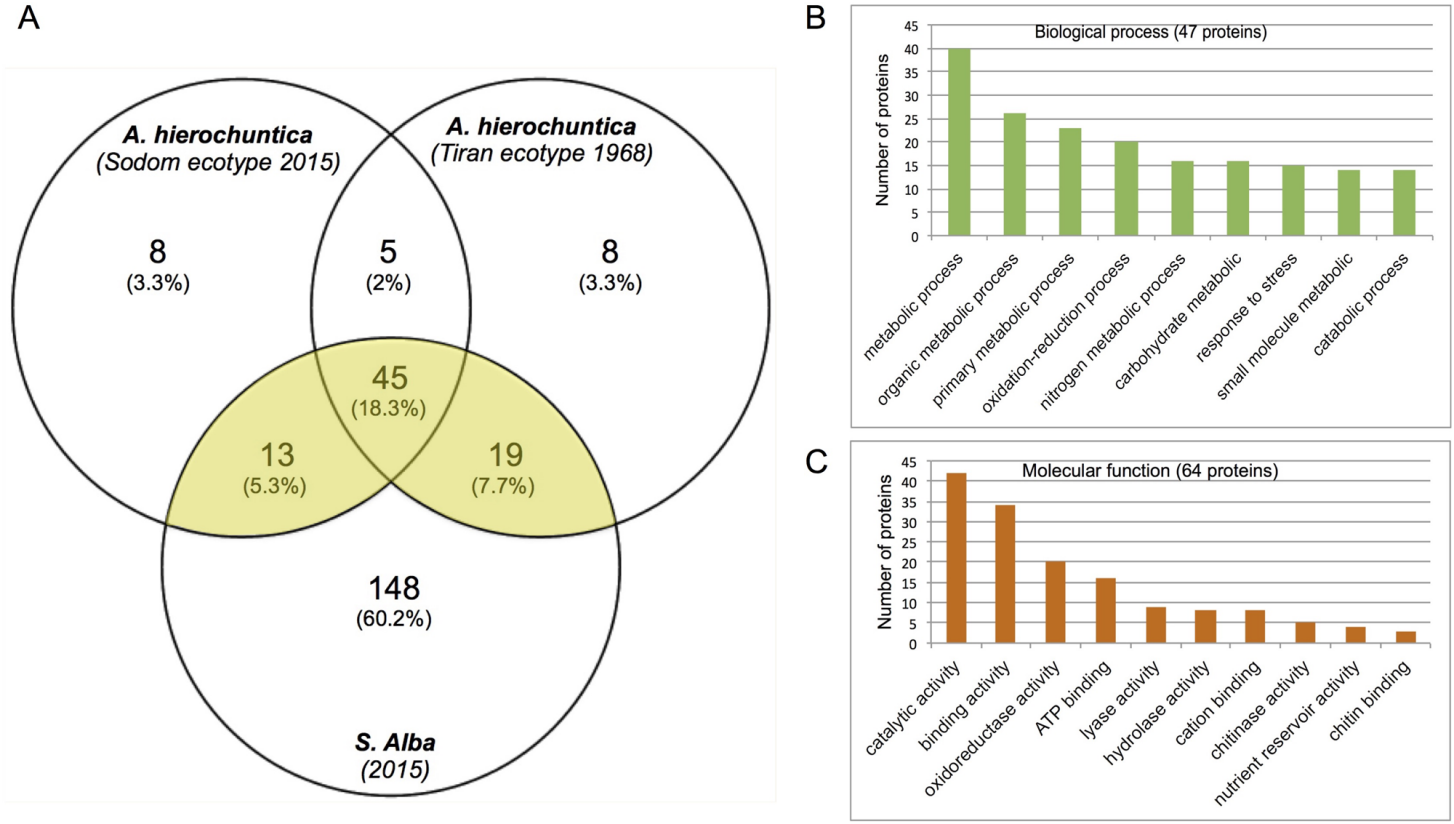
Analysis of proteins released from the dead seed coats of *A*. *hierochuntica* and *S*. *alba*. (A) Venn diagram showing the number of proteins recovered from the dead seed coats of each species examined and the number of proteins shared between the indicated species. 77 proteins shared by *Anastatica* ecotypes and *S*. *alba* highlighted yellow. (B and C) GO categorization for biological process and molecular function, respectively, of the 77 proteins shared by all species examined.

It appears that seed coats of various species function as a major storage compartment for hydrolases such as nucleases and chitinases. We sought to identify proteins specifically released from the seed coat following hydration while attempting to address two aspects, namely, differences between *A*. *hierochuntica* ecotypes new and old collections and differences between different genotypes *A*. *hierochuntica* versus *S*. *alba*. To this end, we compared the proteome profiles of dead seed coats of *A*. *hierochuntica* (Sodom ecotype 2015) and *A*. *hierochuntica* (Tiran Island 1968) and that of *S*. *alba* (2015). Thus, proteins released from seed coats of *A*. *hierochuntica* (2 replicates each ecotype) and *S*. *alba* (3 replicates) were subjected to proteome analysis followed by identification by Discoverer software against the Uniprot database, which contains plants and fungi proteins; only plant proteins were identified (S2 data). Implementing the cutoffs described in Material and methods we identified 246 proteins that are present in at least one plant (S3 data). Accordingly, 71 proteins were identified in Sodom ecotype (2015), 77 in Tiran Island ecotype (1968) and 145 proteins were identified in seed coat of *S*. *alba* (Fig 5A). Venn diagram also shows that 45 proteins are shared by all species/ecotypes examined and 50 proteins out of 98 are shared by *Anastatica* ecotypes Sodom (2015) and Tiran (1968). Notably, out of 98 proteins identified in both *Anastatica* ecotypes, 77 proteins were also released from *S*. *alba* seed coat (S3 data). Functional categorization of these 77 proteins revealed that among the 47 proteins recognized in biological process category, 40 proteins are involved in metabolic processes, 20 in oxidation-reduction processes and 15 proteins are related to response to stress (Fig 5B). Molecular function analysis (Fig 5C) revealed that among the 64 proteins recognized in this category, 42 proteins have catalytic activity including lyase activity (9 proteins), oxireductase activity (8 proteins) and hydrolases (8 proteins including chitinases, peptidases and lipases).

### Analysis of microbial growth controlling activity

**Fig 6:**
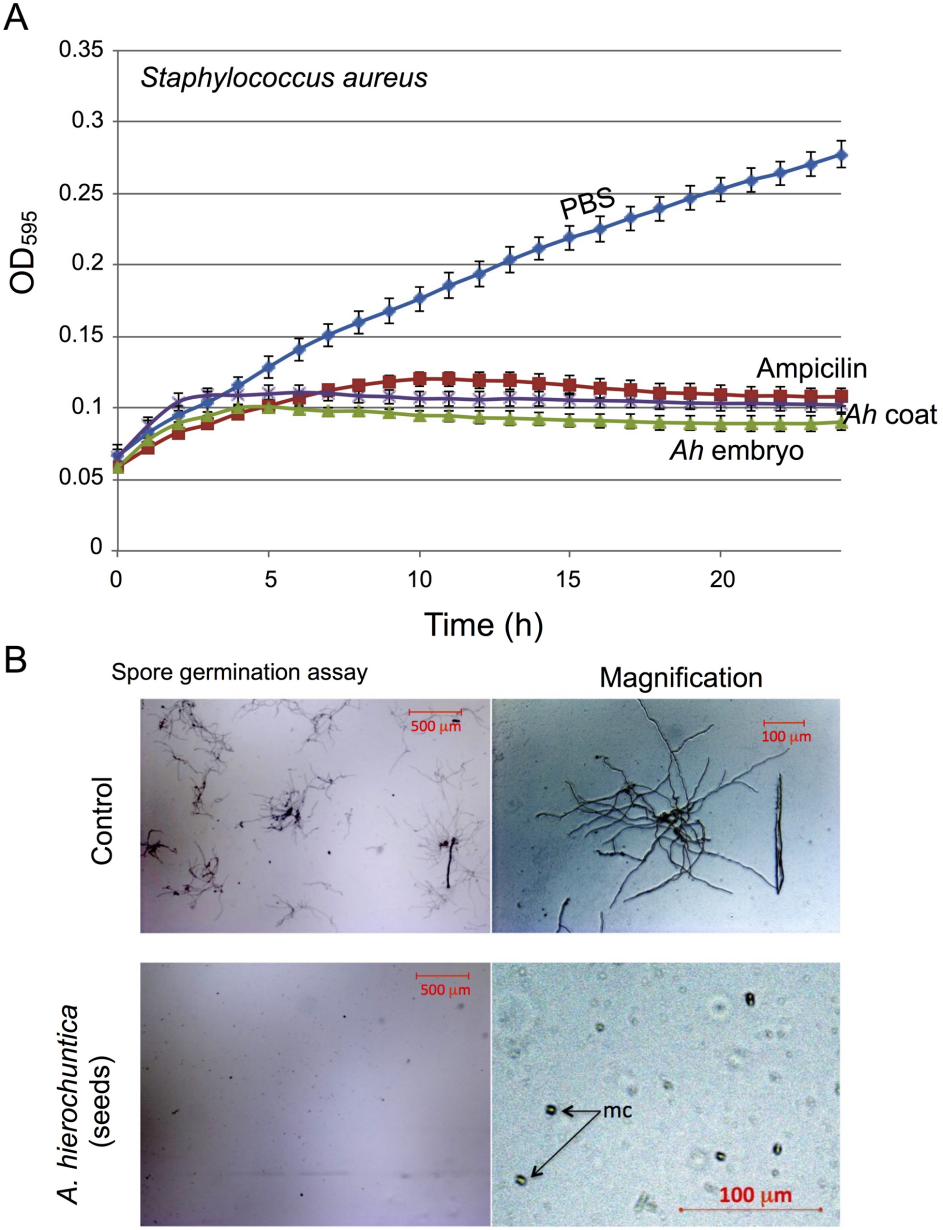
Seeds of the desert plant *Anastatica hierochuntica* release antimicrobial substances. A, *Staphylococcus aureus* was grown in a flat-bottom 96-well microtiter plate in the presence of PBS, ampicillin (100 mg/L) or in the presence of substances released from embryos (*Ah embryo*) or seed coats (*Ah coat*) of *A*. *hierochuntica*. Bacterial growth was monitored by measuring the OD_595_ of the culture at 30 min. intervals in the course of 24 h. Each treatment was performed in triplicates and error bars represent the standard deviation. B, *A*. *hierochuntica* seeds released substances that inhibit conidiospore germination. Microconidia derived from *Fusarium oxysporum* f.sp. *melonis* were mixed with potato dextrose broth (PDB) alone (control) or with PDB supplemented with *A*. *hierochuntica* seed released substances. Mixtures were placed on depression slides and incubated in moist chamber for 24 h at 25°C in a dark and inspected under a light microscope. mc, microconidia.

*A*. *hierochuntica* is commonly used in traditional medicine [50] and the methanolic extract of the plant was reported to contain antimicrobial activity [51]. These prompted us to investigate whether *Anastatica* seeds also store antimicrobial substances that are released upon hydration and what is the origin of these substances, the embryo or the seed coat. We used the Gram-positive strain *Staphylococcus aureus* for antibacterial study. *S*. *aureus* was grown in a flat-bottom 96-well microtiter plate in LB medium supplemented with PBS, ampicillin or with substances released from seed coats or embryos of *A*. *hierochuntica*. Results showed (Fig 6A) that strong antibacterial activity is released from both *A*. *hierochuntica* embryos (*Ah* embryo) and seed coats (*Ah* coat), which was comparable to the inhibitory effect of ampicillin (100 μg/ml). We also found that substances released from intact seeds of *A*. *hierochuntica* inhibited conidial germination of the pathogenic fungus *Fusarium oxysporum* f.sp. *melonis* (Fig 6B). Notably, seeds collected at 1968 from the islands of Tiran and Sanafir, similarly released substances with a strong antibacterial activity.

## Discussion

The seed coat and other hardened parts enclosing the embryo are commonly considered as a passive barrier protecting the embryo from harmful environmental conditions. Here we showed that the dead organ enclosing embryos functions as a storage for active hydrolases and antimicrobial substances that might play important role in regulating seed longevity, germination and seedling establishment. Indeed, the seed coat has been implicated in dormancy and seed longevity and quality [52] and some reports have also suggested the function of the seed coat in storing antimicrobial substances such as tannins, flavonoids and anti fungal proteins that may protect the embryo and germinating seeds from pathogens [27,53].

In the present work, proteome analyses of dead seed coat of *S*. *alba and A*. *hierochuntica* revealed multiple proteins being released upon hydration. Many of these proteins are related to stress response and host defense and include hydrolases (chitinases, proteases and nucleases) and antifungal proteins. Interestingly, the proteome data revealed that the two *Anastatica* ecotypes are clustered together sharing 50 proteins out of the 98 identified in both ecotypes. Importantly, the long-term storage within the dead seed coat of *Anastatica* Tiran ecotype (collected at 1968) not only did not affect mass measurement and protein identification but also had no significant effect on enzymatic activities as demonstrated by in gel nuclease assays. Furthermore, it appears that storage of proteins within the dead seed coat is a general phenomenon in plant seed biology; 78% of the proteins found in *Anastatica* ecotypes (77 proteins) were also found in *S*. *alba* seed coat. Functional categorization of these proteins revealed overrepresentation of proteins related to stress response including three cysteine-rich antifungal proteins related to *Arabidopsis* PDF1.1/LCR67 (At1G75830) and PDF1.2b (AT2G26020). Cystein-rich antifungal proteins are small proteins known as plant defensins [22] that are present in all plant families and can confer enhanced resistance to pathogen when overexpressed in transgenic plants [22,54]. These proteins were primarily found in seeds but are also present in leaves and flowers and often upregulated following pathogenic attack or in response to environmental stress such as drought [55]. Notably, the proteome data revealed xyloglucan endotransglucosylase/hydrolase (XTH), a xyloglucan modifying enzyme, which is thought to play a key role in fruit ripening by loosening the cell wall [56]. This enzyme may assist in loosening of cell walls of the testa and endosperm to allow their rupture and consequently radicle protrusion. This is supported by the findings that in tomato, expression of the XTH gene, SlXET4 was induced by gibberellic acid (GA) in the micropylar endosperm (a region restricting radicle protrusion) during germination [57]. Also, in *Lepidium sativum* the analysis of putative cell-wall-loosening genes (expansins and XTHs) showed that their transcripts are accumulated to high level in the micropylar endosperm 8 h after imbibition [58]. However, in contrast with tomato germinating seeds, in *L*. *sativum* seeds, GA significantly reduced abundance of XTH18 and XTH19 in the micropylar endosperm [58]. Other proteins identified include heat shock proteins HSP70T-1 (related to At1g51090), which is involved in plant immunity and HSP70B (related to At1g16030), peroxidase 12 (related to Arabidopsis At1G71695), as well as chitinases and endochitinases involved in response to pathogens [59,60]. We also identified storage proteins cruciferins that suggest that the seed coat may also contain remnants of the dead endosperm tissue.

Focusing on nucleases, we showed that dead seed coats possess highly active DNases and RNases that can persist in active forms for decades. Some of the DNases appeared to be single stranded DNA endonucleases that are belong to the S1-type endonucleases. This was confirmed by their capacity to convert supercoiled plasmid DNA into relaxed and linear forms, a characteristic feature of S1-type endonucleases such as *Aspergillus* S1 and mung bean nucleases [34,40]. The significance of these nucleases for seed germination and seedling establishment is yet unknown. Endonucleases in general have been implicated in diverse cellular processes including DNA synthesis and DNA repair [61] (Balakrishnan and Bambara, 2013) as well as in fragmentation of genomic DNA during PCD [62–64]. Endonucleases associated with programmed cell death both in plants and animals require Ca^++^ and Mg^++^ for activity [65,66], which is consistent with the requirement of these cations for activity of endonucleases released from seed coats of various crucifers. The capacity of endonucleases to target unpaired regions within superhelical DNA to introduce nicks and DSBs may implicate them as seed defense factors against plasmid-containing soil pathogens. For example, the *Clavibacter michiganensis subsp*. *michiganensis*, a Solanaceae species-pathogenic Gram-positive actinomycete contains two plasmids, designated pCM1 and pCM2, which are important for its pathogenic activity, inasmuch as plasmid-free derivative CMM100 can colonize tomato, but showing no disease symptoms [67]. Targeting these superhelical plasmids by endonucleases released from seeds can lead to neutralization of virulent genes and conversion of a potentially pathogen into a non-pathogenic one. Also, RNases were shown to inhibit growth of pathogenic fungi. Accordingly, exogenous application of S-like RNase NE into the extracellular space of leaves inhibits the development of *Phytophthora parasitica* [68,69], a oomycete soilborne pathogen with a wide range of host plants. Also, the Wheatwin1 PR4 RNase was shown to enter inside fungal cells without affecting the integrity of cell walls and possesses antifungal activity, which is dependent on its enzymatic activity [70]. In-gel RNase assays showed the activity of multiple RNAses released from the seed coat and the embryo of various Brassicaceae species further highlighting the wide range of released molecules that have the potential to act as pathogen inhibitors. Notably, the proteome analysis revealed multiple proteins that could act against pathogens including chitinases and endochitinases. Chitinases are enzymes that degrade chitin an abundant polysaccharide found in a variety of organisms including insects, fungi, yeast, and algae. Chitinase and glucanase genes were often over-expressed in plants to confer resistance against fungal pathogens [71–73].

Proteome analysis was performed for the seed coat (testa) of soybean (*Glycine max* L. Merr. cv Jack) during various stages of seed development [74]. Interestingly, the proteins extracted from soybean seed coat were comparable to proteins released upon hydration from *Arabidopsis* mature seeds as well as from seed coats of *Anastatica hierochuntica* and *Sinapis alba*. In all cases, hundreds of proteins were identified and categorized into several functional groups including metabolic processes and stress response. The over-representation of proteins involved in stress response (Fig 5B), suggests that these proteins are accumulated in the dead seed coats during seed development and might provide an additional active defense layer for embryo protection.

Presently, we could not find a clear correlation between hydrolase activities and inhibition of bacterial growth directed by substances released from *A*. *hierochuntica*. Accordingly, both embryo and seed coats released strong antimicrobial activity, yet nucleases were released exclusively from the seed coat suggesting that nucleases may not be the principal inhibitory factor(s) of bacterial growth. Alternatively, a metabolite or combination of metabolites may provide the principal factor(s) inhibiting bacterial growth. Many such metabolites with antimicrobial activities exist in plants including alkaloids, phenolic compounds and terpenoids [75].

## Conclusions

The finding that proteins are stored and remained active within essentially dead organs of the seed coat for many years is puzzling. It is commonly believed that cellular proteins undergo complete degradation when cell die and their constituents are remobilized to other parts of the plants (young leaves, fruits, embryos). Based on the data presented here and in other report [31] we suggest that seed coat and also other dead hardened parts enclosing embryos (e.g., glumes, lemmas and palease in Poaceae species, [31]) were evolved not just for providing a physical shield for embryo protection but also as storage organs for multiple active proteins and probably metabolites and other substances for the purpose of nourishment as well as protection of germinating seeds from soil pathogens, which may facilitate seed persistence in the soil, germination and seedling establishment in wild as well as in agroecosystem [76].

Considering the sessile nature of plants and that the seed coat is a maternally derived organ, our future goal is to explore how exposure of mother plants to biotic and abiotic stresses during flowering and seed maturation affects the composition and activities (e.g., hydrolase activities, antimicrobial activities) of substances stored in and released from seed coats upon hydration as well as the effect of the seed coat on seed germination and seedling establishment. The realization that plant dead organs store active molecules and possibly multiple beneficial substances might change the way we treat and refer to plant remnants in agricultural practices as well as the way we store seeds in seed banks.

## Supporting information

S1 File Figure A. Functional classification of released proteins from Arabidopsis (Col) seeds. Figure B. Timing of release of nucleases from the *S*. *alba* seeds following hydration. Figure C. Nuclease activities released from seed coats of various leguminous plants. S1 Table. A list of plant species used in the present study. S2 Table. Proteome parameter definition. S1 data. Proteome raw data of proteins released from *Arabidopsis thaliana* seeds upon hydration. S2 data. Proteome raw data proteins released from seed coats of *Sinapis alba* and *Anastatica hierochuntica*. S3 data. List of proteins released from dead seed coats shared by *Sinapis alba* and *Anastatica hierochuntica*.
(PDF)
