# Supplementary material for: The dead seed coat functions as a long-term storage for active hydrolytic enzymes
Source: PLoS One. 2017 Jul 11;12(7):e0181102. doi: 10.1371/journal.pone.0181102 (PMC5507414; doi:10.1371/journal.pone.0181102)
Supplement: S1 File — Figure A. Functional classification of released proteins from Arabidopsis (Col) seeds. Figure B. Timing of release of nucleases from the S. alba seeds following hydration. Figure C. Nuclease activities released from seed coats of various leguminous plants. S1 Table. A list of plant species used in the present study. S2 Table. Proteome parameter definition. S1 data. Proteome raw data of proteins released from Arabidopsis thaliana seeds upon hydration. S2 data. Proteome raw data proteins released from seed coats of Sinapis alba and Anastatica hierochuntica. S3 data. List of proteins released from dead seed coats shared by Sinapis alba and Anastatica hierochuntica. (PDF) [file pone.0181102.s001.pdf]

Supplementary materials

The Dead Seed Coat Functions as a Long-Term Storage for Active Hydrolytic Enzymes

Buzi Raviv[1], Lusine Aghajanyan [1], Gila Granot [1], Vardit Makover [2], Omer Frenkel [3], Yitzchak Gutterman [1] and Gideon Grafi [1]*

[1]French Associates Institute of Agriculture and Biotechnology of Drylands, The Institutes for Desert Research, Ben-Gurion University of the Negev, Midreshet Ben-Gurion, 84990, Israel; [2]The Zuckerberg Institute for Water Research, The Institutes for Desert Research, Ben-Gurion University of the Negev, Midreshet Ben-Gurion, 84990, Israel; [3]Department of Plant Pathology and Weed Research, ARO, The Volcani Center, Bet Dagan 50250, Israel

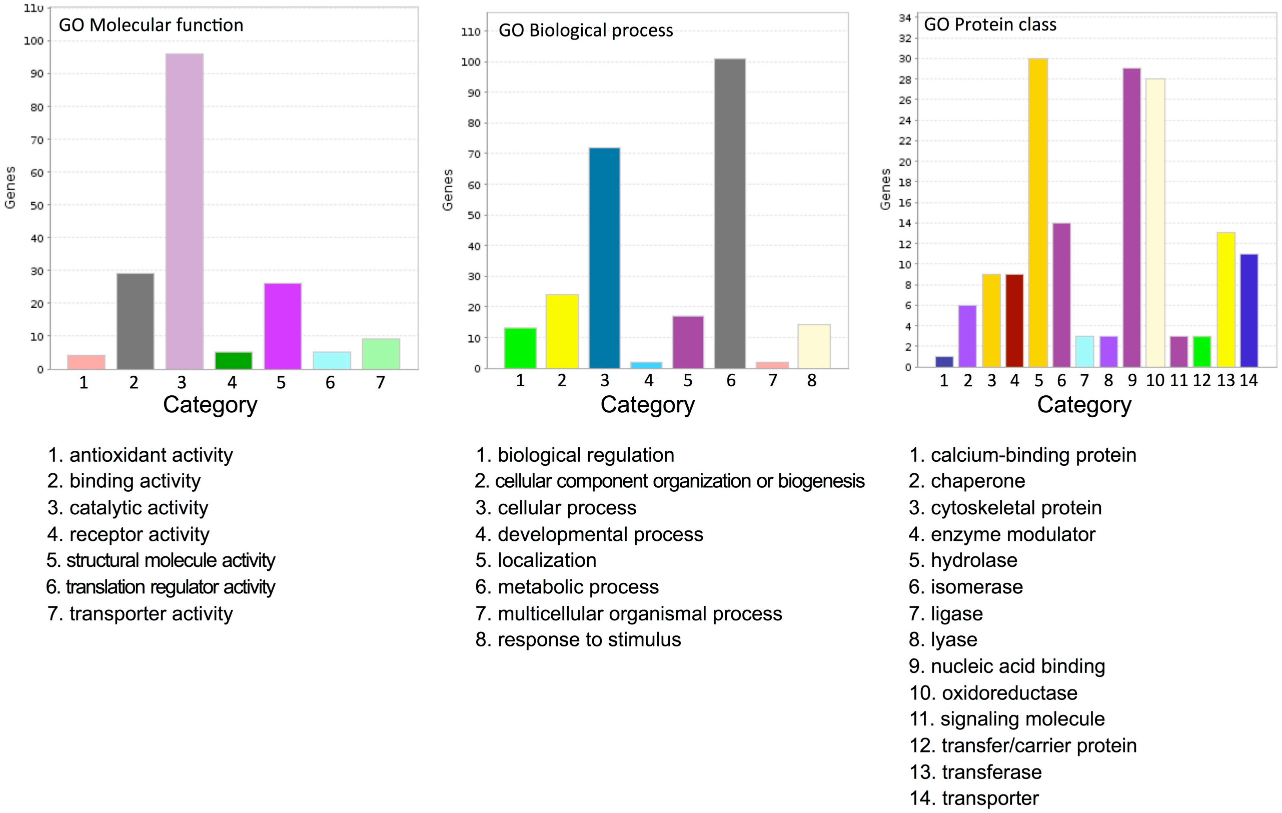

Figure A. Functional classification of secreted proteins from Arabidopsis (Col) seeds following hydration (PBS) at 4°C for 12 h. Classification was performed using PANTHER classification system (Mi et al., 2005; http://pantherdb.org).

Mi H, Lazareva-Ulitsky B, Loo R, Kejariwal A, Vandergriff J, Rabkin S, et al. The PANTHER database of protein families, subfamilies, functions and pathways. Nucleic Acids Research 2005; 33, D284-D288.

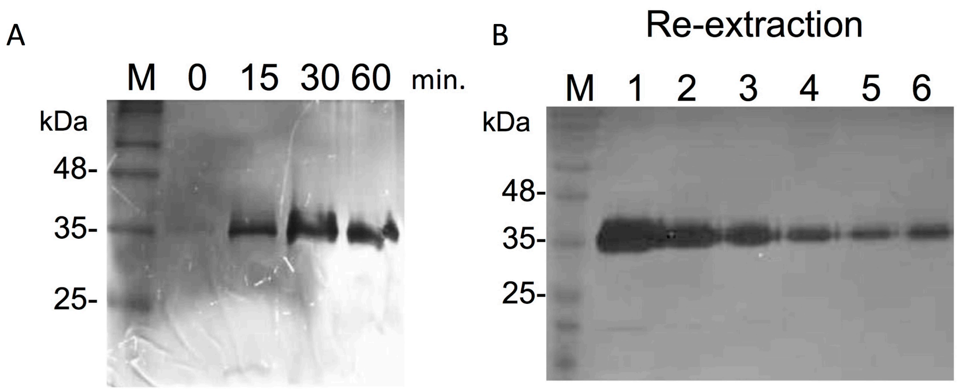

Figure B. Release of nucleases from the *S. alba* and *A. hierochuntica* seeds and seed coats. A, Seeds were incubated in PBS for various time points. The aqueous phase was collected and analyzed by in-gel nuclease assay toward denatured salmon sperm DNA (ssDNA). B, Repeated extraction of nucleases from seed coats. Seed coats of *S. alba* were subjected to five rounds of extraction with PBS at 3h intervals (lanes 1-5). Final extraction (lane 6) was performed by adding PBS for another 9h. Lane M is molecular weight protein markers.

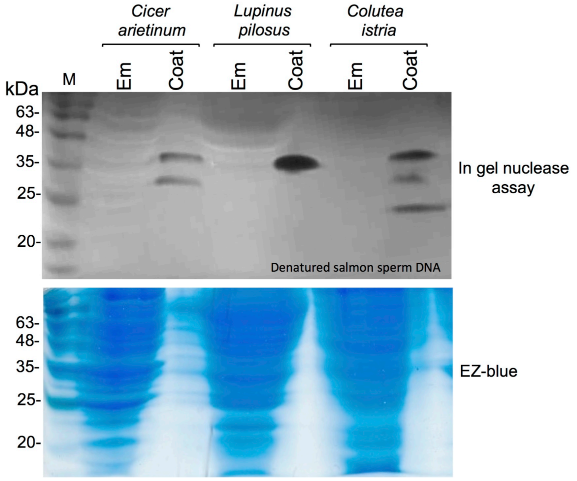

Figure C. The seed coats of leguminous species store and release upon hydration nucleases. Upper panel, Proteins released from seed coats and embryos (Em) of the indicated leguminous species were subjected to in gel nuclease assay using denatured salmon sperm DNA as substrate. Lower panel is the EZBlue staining gel. Note that nuclease activity could not be detected in embryos in spite of the large amount of proteins.
